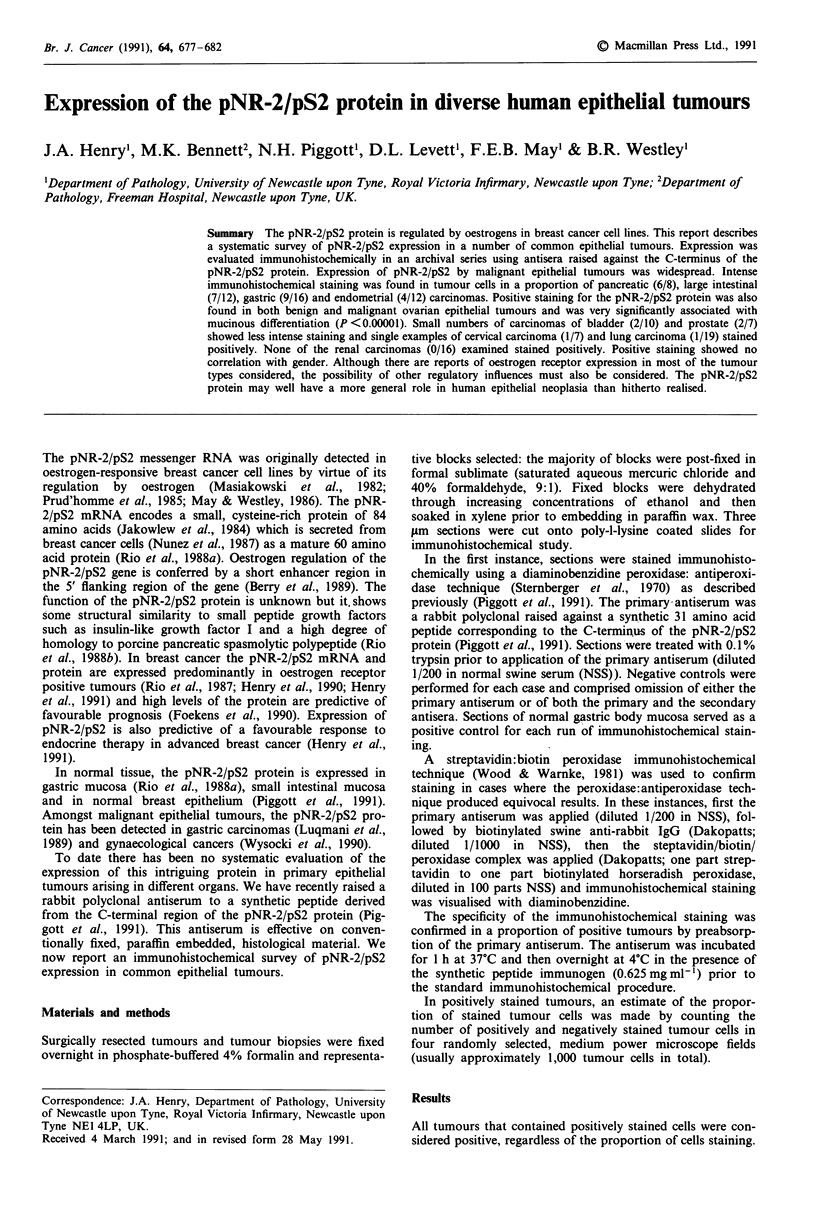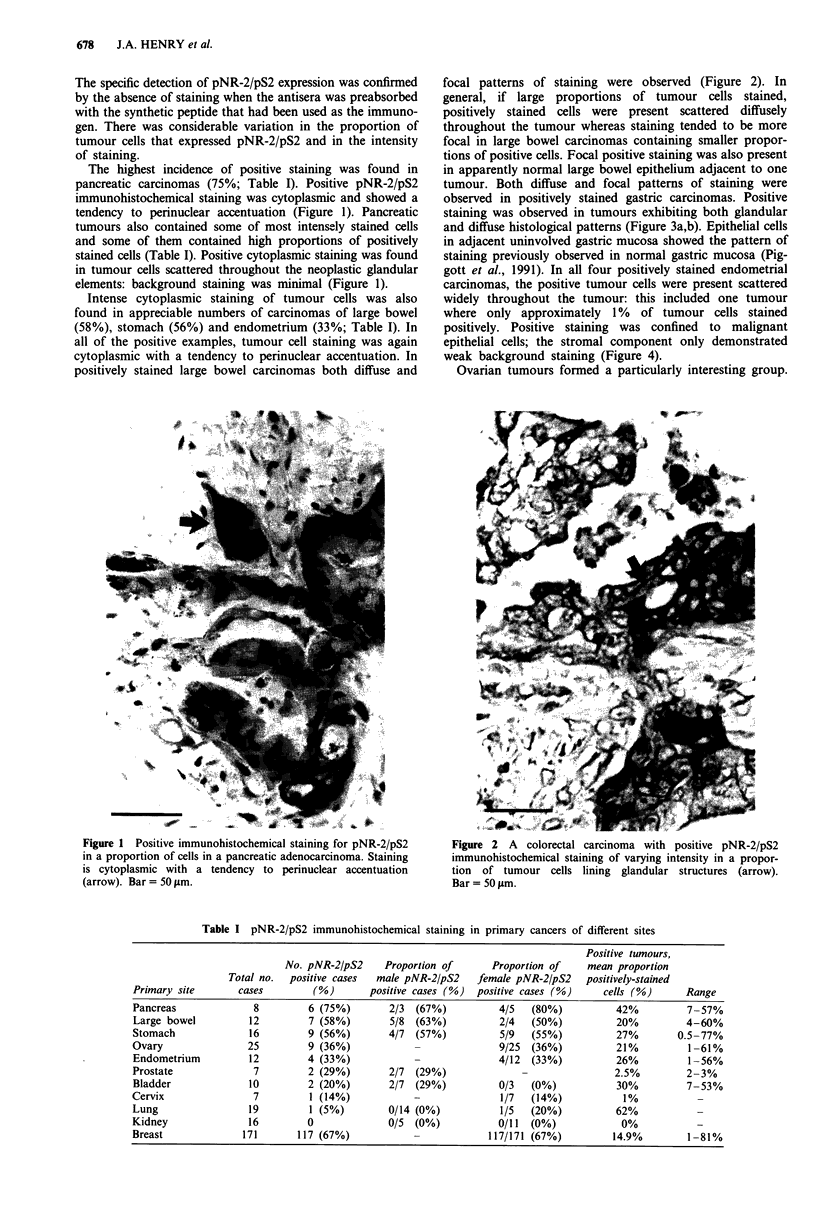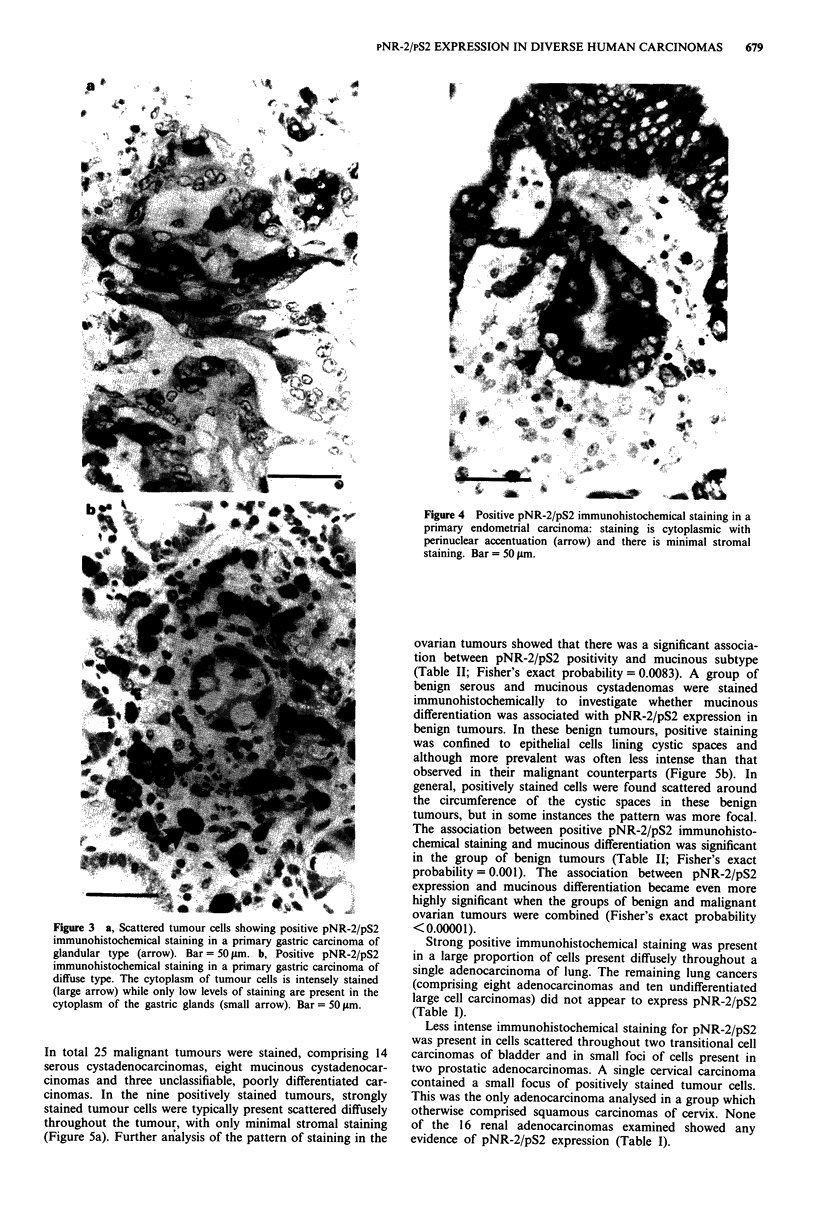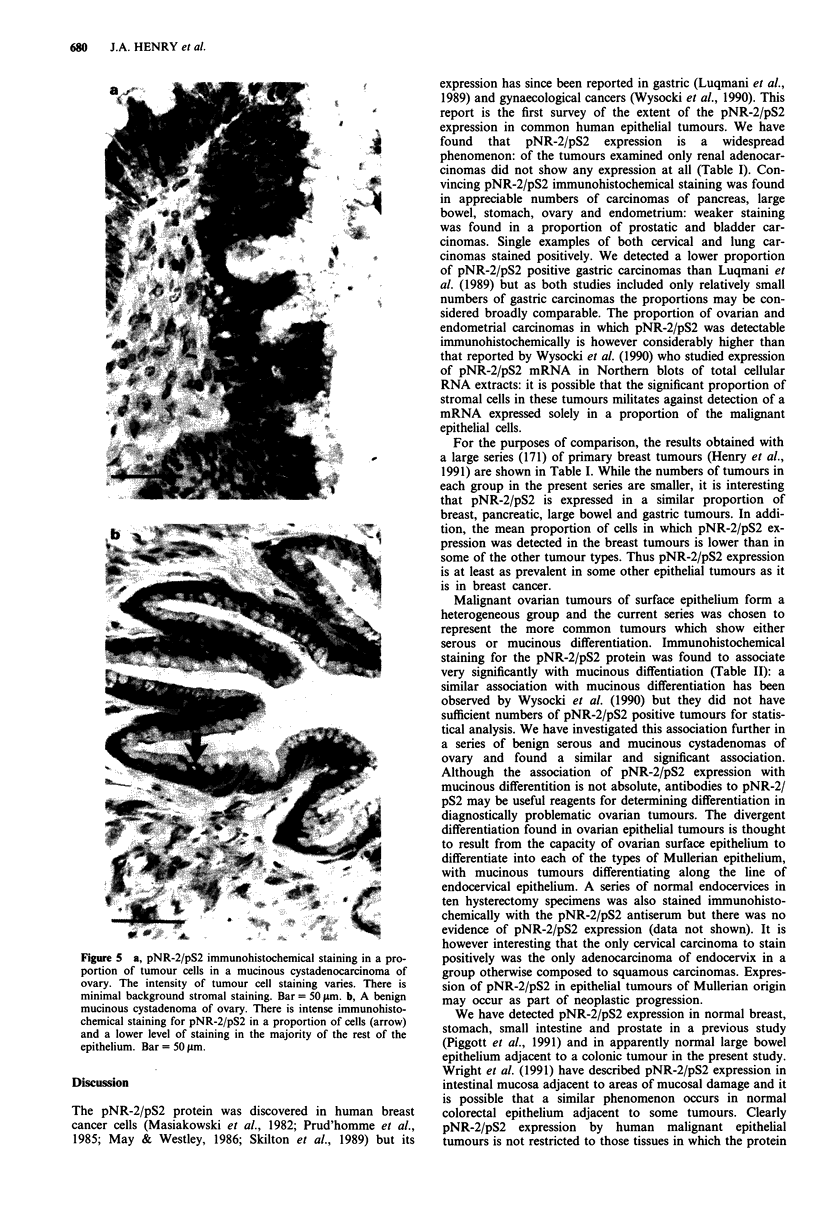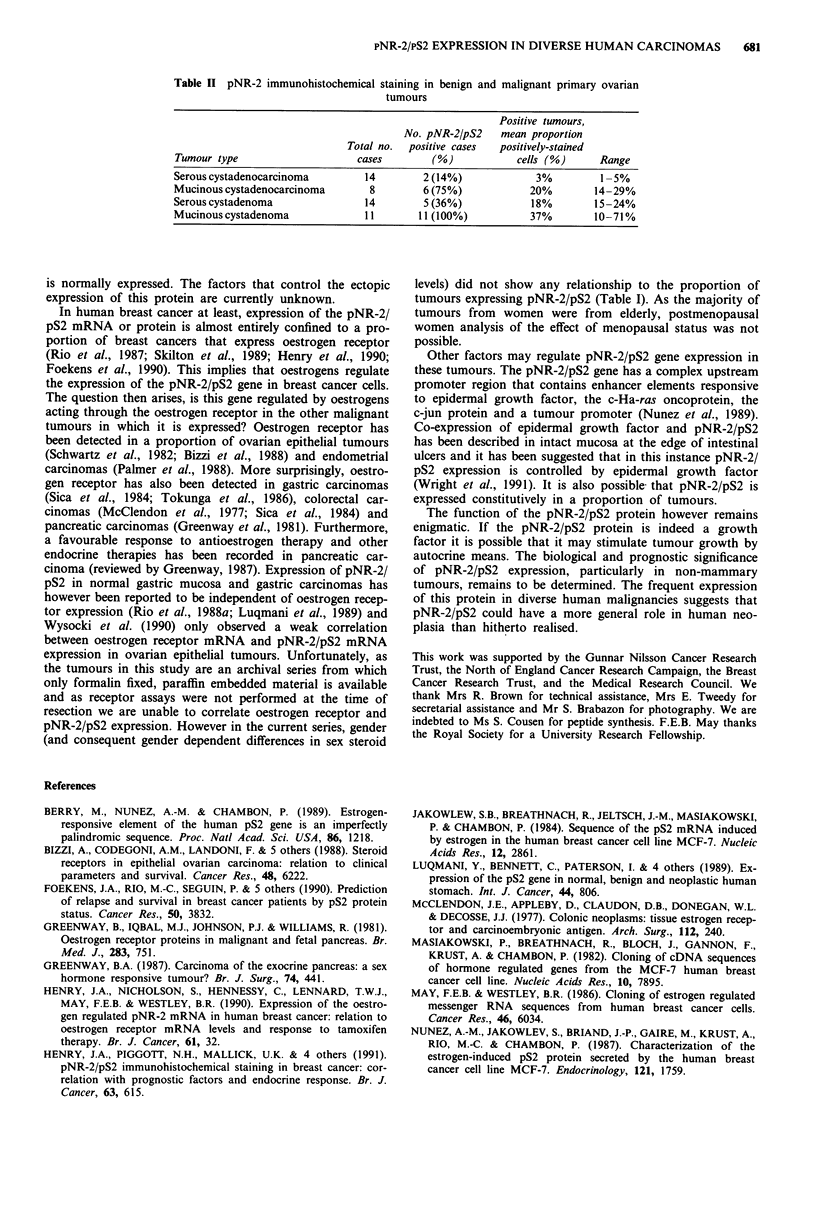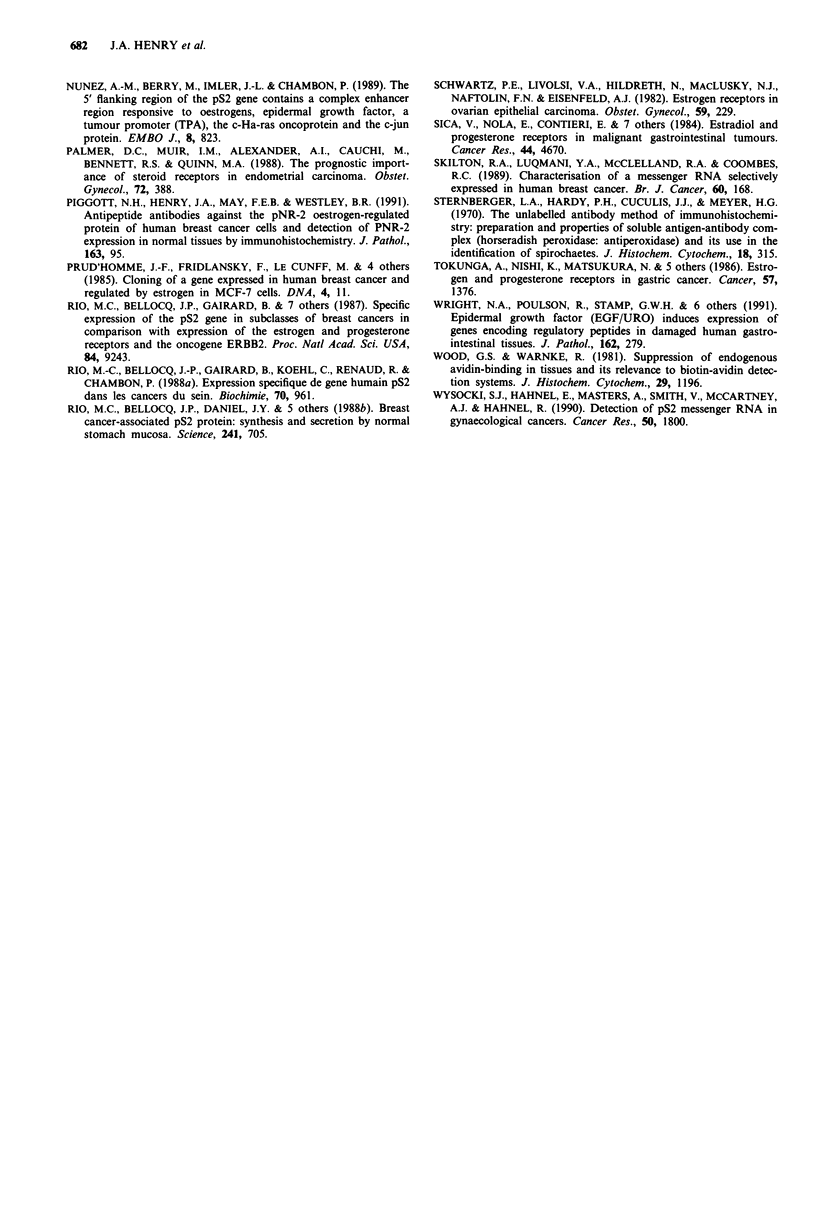# Expression of the pNR-2/pS2 protein in diverse human epithelial tumours.

**DOI:** 10.1038/bjc.1991.380

**Published:** 1991-10

**Authors:** J. A. Henry, M. K. Bennett, N. H. Piggott, D. L. Levett, F. E. May, B. R. Westley

**Affiliations:** Department of Pathology, University of Newcastle upon Tyne, Royal Victoria Infirmary, UK.

## Abstract

**Images:**


Br. J. Cancer (1991), 64, 677-682 © Macmillan Press Ltd., 1991

### Expression of the pNR-2/pS2 protein in diverse human epithelial tumours

J.A. Henry[1], M.K. Bennett[2], N.H. Piggott[1], D.L. Levett[1], F.E.B. May[1] & B.R. Westley[1]

[1]*Department of Pathology, University of Newcastle upon Tyne, Royal Victoria Infirmary, Newcastle upon Tyne;* [2]*Department of Pathology, Freeman Hospital, Newcastle upon Tyne, UK.*

**Summary** The pNR-2/pS2 protein is regulated by oestrogens in breast cancer cell lines. This report describes a systematic survey of pNR-2/pS2 expression in a number of common epithelial tumours. Expression was evaluated immunohistochemically in an archival series using antisera raised against the C-terminus of the pNR-2/pS2 protein. Expression of pNR-2/pS2 by malignant epithelial tumours was widespread. Intense immunohistochemical staining was found in tumour cells in a proportion of pancreatic (6/8), large intestinal (7/12), gastric (9/16) and endometrial (4/12) carcinomas. Positive staining for the pNR-2/pS2 protein was also found in both benign and malignant ovarian epithelial tumours and was very significantly associated with mucinous differentiation ($P < 0.00001$). Small numbers of carcinomas of bladder (2/10) and prostate (2/7) showed less intense staining and single examples of cervical carcinoma (1/7) and lung carcinoma (1/19) stained positively. None of the renal carcinomas (0/16) examined stained positively. Positive staining showed no correlation with gender. Although there are reports of oestrogen receptor expression in most of the tumour types considered, the possibility of other regulatory influences must also be considered. The pNR-2/pS2 protein may well have a more general role in human epithelial neoplasia than hitherto realised.

The pNR-2/pS2 messenger RNA was originally detected in oestrogen-responsive breast cancer cell lines by virtue of its regulation by oestrogen (Masiakowski *et al.*, 1982; Prud'homme *et al.*, 1985; May & Westley, 1986). The pNR-2/pS2 mRNA encodes a small, cysteine-rich protein of 84 amino acids (Jakowlew *et al.*, 1984) which is secreted from breast cancer cells (Nunez *et al.*, 1987) as a mature 60 amino acid protein (Rio *et al.*, 1988*a*). Oestrogen regulation of the pNR-2/pS2 gene is conferred by a short enhancer region in the 5′ flanking region of the gene (Berry *et al.*, 1989). The function of the pNR-2/pS2 protein is unknown but it shows some structural similarity to small peptide growth factors such as insulin-like growth factor I and a high degree of homology to porcine pancreatic spasmolytic polypeptide (Rio *et al.*, 1988*b*). In breast cancer the pNR-2/pS2 mRNA and protein are expressed predominantly in oestrogen receptor positive tumours (Rio *et al.*, 1987; Henry *et al.*, 1990; Henry *et al.*, 1991) and high levels of the protein are predictive of favourable prognosis (Foekens *et al.*, 1990). Expression of pNR-2/pS2 is also predictive of a favourable response to endocrine therapy in advanced breast cancer (Henry *et al.*, 1991).

In normal tissue, the pNR-2/pS2 protein is expressed in gastric mucosa (Rio *et al.*, 1988*a*), small intestinal mucosa and in normal breast epithelium (Piggott *et al.*, 1991). Amongst malignant epithelial tumours, the pNR-2/pS2 protein has been detected in gastric carcinomas (Luqmani *et al.*, 1989) and gynaecological cancers (Wysocki *et al.*, 1990).

To date there has been no systematic evaluation of the expression of this intriguing protein in primary epithelial tumours arising in different organs. We have recently raised a rabbit polyclonal antiserum to a synthetic peptide derived from the C-terminal region of the pNR-2/pS2 protein (Piggott *et al.*, 1991). This antiserum is effective on conventionally fixed, paraffin embedded, histological material. We now report an immunohistochemical survey of pNR-2/pS2 expression in common epithelial tumours.

### Materials and methods

Surgically resected tumours and tumour biopsies were fixed overnight in phosphate-buffered 4% formalin and representative blocks selected: the majority of blocks were post-fixed in formal sublimate (saturated aqueous mercuric chloride and 40% formaldehyde, 9:1). Fixed blocks were dehydrated through increasing concentrations of ethanol and then soaked in xylene prior to embedding in paraffin wax. Three μm sections were cut onto poly-l-lysine coated slides for immunohistochemical study.

In the first instance, sections were stained immunohistochemically using a diaminobenzidine peroxidase: antiperoxidase technique (Sternberger *et al.*, 1970) as described previously (Piggott *et al.*, 1991). The primary antiserum was a rabbit polyclonal raised against a synthetic 31 amino acid peptide corresponding to the C-terminus of the pNR-2/pS2 protein (Piggott *et al.*, 1991). Sections were treated with 0.1% trypsin prior to application of the primary antiserum (diluted 1/200 in normal swine serum (NSS)). Negative controls were performed for each case and comprised omission of either the primary antiserum or of both the primary and the secondary antisera. Sections of normal gastric body mucosa served as a positive control for each run of immunohistochemical staining.

A streptavidin:biotin peroxidase immunohistochemical technique (Wood & Warnke, 1981) was used to confirm staining in cases where the peroxidase:antiperoxidase technique produced equivocal results. In these instances, first the primary antiserum was applied (diluted 1/200 in NSS), followed by biotinylated swine anti-rabbit IgG (Dakopatts; diluted 1/1000 in NSS), then the steptavidin/biotin/peroxidase complex was applied (Dakopatts; one part streptavidin to one part biotinylated horseradish peroxidase, diluted in 100 parts NSS) and immunohistochemical staining was visualised with diaminobenzidine.

The specificity of the immunohistochemical staining was confirmed in a proportion of positive tumours by preabsorption of the primary antiserum. The antiserum was incubated for 1 h at 37°C and then overnight at 4°C in the presence of the synthetic peptide immunogen (0.625 mg $ml^{-1}$) prior to the standard immunohistochemical procedure.

In positively stained tumours, an estimate of the proportion of stained tumour cells was made by counting the number of positively and negatively stained tumour cells in four randomly selected, medium power microscope fields (usually approximately 1,000 tumour cells in total).

### Results

All tumours that contained positively stained cells were considered positive, regardless of the proportion of cells staining.

Correspondence: J.A. Henry, Department of Pathology, University of Newcastle upon Tyne, Royal Victoria Infirmary, Newcastle upon Tyne NE1 4LP, UK.
Received 4 March 1991; and in revised form 28 May 1991.

The specific detection of pNR-2/pS2 expression was confirmed by the absence of staining when the antisera was preabsorbed with the synthetic peptide that had been used as the immunogen. There was considerable variation in the proportion of tumour cells that expressed pNR-2/pS2 and in the intensity of staining.

The highest incidence of positive staining was found in pancreatic carcinomas (75%; Table I). Positive pNR-2/pS2 immunohistochemical staining was cytoplasmic and showed a tendency to perinuclear accentuation (Figure 1). Pancreatic tumours also contained some of most intensely stained cells and some of them contained high proportions of positively stained cells (Table I). Positive cytoplasmic staining was found in tumour cells scattered throughout the neoplastic glandular elements: background staining was minimal (Figure 1).

Intense cytoplasmic staining of tumour cells was also found in appreciable numbers of carcinomas of large bowel (58%), stomach (56%) and endometrium (33%; Table I). In all of the positive examples, tumour cell staining was again cytoplasmic with a tendency to perinuclear accentuation. In positively stained large bowel carcinomas both diffuse and focal patterns of staining were observed (Figure 2). In general, if large proportions of tumour cells stained, positively stained cells were present scattered diffusely throughout the tumour whereas staining tended to be more focal in large bowel carcinomas containing smaller proportions of positive cells. Focal positive staining was also present in apparently normal large bowel epithelium adjacent to one tumour. Both diffuse and focal patterns of staining were observed in positively stained gastric carcinomas. Positive staining was observed in tumours exhibiting both glandular and diffuse histological patterns (Figure 3a,b). Epithelial cells in adjacent uninvolved gastric mucosa showed the pattern of staining previously observed in normal gastric mucosa (Piggott et al., 1991). In all four positively stained endometrial carcinomas, the positive tumour cells were present scattered widely throughout the tumour: this included one tumour where only approximately 1% of tumour cells stained positively. Positive staining was confined to malignant epithelial cells; the stromal component only demonstrated weak background staining (Figure 4).

Ovarian tumours formed a particularly interesting group.

**Figure 1** Positive immunohistochemical staining for pNR-2/pS2 in a proportion of cells in a pancreatic adenocarcinoma. Staining is cytoplasmic with a tendency to perinuclear accentuation (arrow). Bar = 50 μm.

**Figure 2** A colorectal carcinoma with positive pNR-2/pS2 immunohistochemical staining of varying intensity in a proportion of tumour cells lining glandular structures (arrow). Bar = 50 μm.

**Table I** pNR-2/pS2 immunohistochemical staining in primary cancers of different sites

| *Primary site* | *Total no. cases* | *No. pNR-2/pS2 positive cases (%)* | *Proportion of male pNR-2/pS2 positive cases (%)* | *Proportion of female pNR-2/pS2 positive cases (%)* | *Positive tumours, mean proportion positively-stained cells (%)* | *Range* |
|---|---|---|---|---|---|---|
| Pancreas | 8 | 6 (75%) | 2/3 (67%) | 4/5 (80%) | 42% | 7–57% |
| Large bowel | 12 | 7 (58%) | 5/8 (63%) | 2/4 (50%) | 20% | 4–60% |
| Stomach | 16 | 9 (56%) | 4/7 (57%) | 5/9 (55%) | 27% | 0.5–77% |
| Ovary | 25 | 9 (36%) | – | 9/25 (36%) | 21% | 1–61% |
| Endometrium | 12 | 4 (33%) | – | 4/12 (33%) | 26% | 1–56% |
| Prostate | 7 | 2 (29%) | 2/7 (29%) | – | 2.5% | 2–3% |
| Bladder | 10 | 2 (20%) | 2/7 (29%) | 0/3 (0%) | 30% | 7–53% |
| Cervix | 7 | 1 (14%) | – | 1/7 (14%) | 1% | – |
| Lung | 19 | 1 (5%) | 0/14 (0%) | 1/5 (20%) | 62% | – |
| Kidney | 16 | 0 | 0/5 (0%) | 0/11 (0%) | 0% | – |
| Breast | 171 | 117 (67%) | – | 117/171 (67%) | 14.9% | 1–81% |

**Figure 3** **a**, Scattered tumour cells showing positive pNR-2/pS2 immunohistochemical staining in a primary gastric carcinoma of glandular type (arrow). Bar = 50 μm. **b**, Positive pNR-2/pS2 immunohistochemical staining in a primary gastric carcinoma of diffuse type. The cytoplasm of tumour cells is intensely stained (large arrow) while only low levels of staining are present in the cytoplasm of the gastric glands (small arrow). Bar = 50 μm.

**Figure 4** Positive pNR-2/pS2 immunohistochemical staining in a primary endometrial carcinoma: staining is cytoplasmic with perinuclear accentuation (arrow) and there is minimal stromal staining. Bar = 50 μm.

In total 25 malignant tumours were stained, comprising 14 serous cystadenocarcinomas, eight mucinous cystadenocarcinomas and three unclassifiable, poorly differentiated carcinomas. In the nine positively stained tumours, strongly stained tumour cells were typically present scattered diffusely throughout the tumour, with only minimal stromal staining (Figure 5a). Further analysis of the pattern of staining in the ovarian tumours showed that there was a significant association between pNR-2/pS2 positivity and mucinous subtype (Table II; Fisher's exact probability = 0.0083). A group of benign serous and mucinous cystadenomas were stained immunohistochemically to investigate whether mucinous differentiation was associated with pNR-2/pS2 expression in benign tumours. In these benign tumours, positive staining was confined to epithelial cells lining cystic spaces and although more prevalent was often less intense than that observed in their malignant counterparts (Figure 5b). In general, positively stained cells were found scattered around the circumference of the cystic spaces in these benign tumours, but in some instances the pattern was more focal. The association between positive pNR-2/pS2 immunohistochemical staining and mucinous differentiation was significant in the group of benign tumours (Table II; Fisher's exact probability = 0.001). The association between pNR-2/pS2 expression and mucinous differentiation became even more highly significant when the groups of benign and malignant ovarian tumours were combined (Fisher's exact probability $<0.00001$).

Strong positive immunohistochemical staining was present in a large proportion of cells present diffusely throughout a single adenocarcinoma of lung. The remaining lung cancers (comprising eight adenocarcinomas and ten undifferentiated large cell carcinomas) did not appear to express pNR-2/pS2 (Table I).

Less intense immunohistochemical staining for pNR-2/pS2 was present in cells scattered throughout two transitional cell carcinomas of bladder and in small foci of cells present in two prostatic adenocarcinomas. A single cervical carcinoma contained a small focus of positively stained tumour cells. This was the only adenocarcinoma analysed in a group which otherwise comprised squamous carcinomas of cervix. None of the 16 renal adenocarcinomas examined showed any evidence of pNR-2/pS2 expression (Table I).

**Figure 5** **a**, pNR-2/pS2 immunohistochemical staining in a proportion of tumour cells in a mucinous cystadenocarcinoma of ovary. The intensity of tumour cell staining varies. There is minimal background stromal staining. Bar = 50 μm. **b**, A benign mucinous cystadenoma of ovary. There is intense immunohistochemical staining for pNR-2/pS2 in a proportion of cells (arrow) and a lower level of staining in the majority of the rest of the epithelium. Bar = 50 μm.

### Discussion

The pNR-2/pS2 protein was discovered in human breast cancer cells (Masiakowski *et al.*, 1982; Prud'homme *et al.*, 1985; May & Westley, 1986; Skilton *et al.*, 1989) but its expression has since been reported in gastric (Luqmani *et al.*, 1989) and gynaecological cancers (Wysocki *et al.*, 1990). This report is the first survey of the extent of the pNR-2/pS2 expression in common human epithelial tumours. We have found that pNR-2/pS2 expression is a widespread phenomenon: of the tumours examined only renal adenocarcinomas did not show any expression at all (Table I). Convincing pNR-2/pS2 immunohistochemical staining was found in appreciable numbers of carcinomas of pancreas, large bowel, stomach, ovary and endometrium: weaker staining was found in a proportion of prostatic and bladder carcinomas. Single examples of both cervical and lung carcinomas stained positively. We detected a lower proportion of pNR-2/pS2 positive gastric carcinomas than Luqmani *et al.* (1989) but as both studies included only relatively small numbers of gastric carcinomas the proportions may be considered broadly comparable. The proportion of ovarian and endometrial carcinomas in which pNR-2/pS2 was detectable immunohistochemically is however considerably higher than that reported by Wysocki *et al.* (1990) who studied expression of pNR-2/pS2 mRNA in Northern blots of total cellular RNA extracts: it is possible that the significant proportion of stromal cells in these tumours militates against detection of a mRNA expressed solely in a proportion of the malignant epithelial cells.

For the purposes of comparison, the results obtained with a large series (171) of primary breast tumours (Henry *et al.*, 1991) are shown in Table I. While the numbers of tumours in each group in the present series are smaller, it is interesting that pNR-2/pS2 is expressed in a similar proportion of breast, pancreatic, large bowel and gastric tumours. In addition, the mean proportion of cells in which pNR-2/pS2 expression was detected in the breast tumours is lower than in some of the other tumour types. Thus pNR-2/pS2 expression is at least as prevalent in some other epithelial tumours as it is in breast cancer.

Malignant ovarian tumours of surface epithelium form a heterogeneous group and the current series was chosen to represent the more common tumours which show either serous or mucinous differentiation. Immunohistochemical staining for the pNR-2/pS2 protein was found to associate very significantly with mucinous diffentiation (Table II): a similar association with mucinous differentiation has been observed by Wysocki *et al.* (1990) but they did not have sufficient numbers of pNR-2/pS2 positive tumours for statistical analysis. We have investigated this association further in a series of benign serous and mucinous cystadenomas of ovary and found a similar and significant association. Although the association of pNR-2/pS2 expression with mucinous differentition is not absolute, antibodies to pNR-2/pS2 may be useful reagents for determining differentiation in diagnostically problematic ovarian tumours. The divergent differentiation found in ovarian epithelial tumours is thought to result from the capacity of ovarian surface epithelium to differentiate into each of the types of Mullerian epithelium, with mucinous tumours differentiating along the line of endocervical epithelium. A series of normal endocervices in ten hysterectomy specimens was also stained immunohistochemically with the pNR-2/pS2 antiserum but there was no evidence of pNR-2/pS2 expression (data not shown). It is however interesting that the only cervical carcinoma to stain positively was the only adenocarcinoma of endocervix in a group otherwise composed to squamous carcinomas. Expression of pNR-2/pS2 in epithelial tumours of Mullerian origin may occur as part of neoplastic progression.

We have detected pNR-2/pS2 expression in normal breast, stomach, small intestine and prostate in a previous study (Piggott *et al.*, 1991) and in apparently normal large bowel epithelium adjacent to a colonic tumour in the present study. Wright *et al.* (1991) have described pNR-2/pS2 expression in intestinal mucosa adjacent to areas of mucosal damage and it is possible that a similar phenomenon occurs in normal colorectal epithelium adjacent to some tumours. Clearly pNR-2/pS2 expression by human malignant epithelial tumours is not restricted to those tissues in which the protein

**Table II** pNR-2 immunohistochemical staining in benign and malignant primary ovarian tumours

| *Tumour type* | *Total no. cases* | *No. pNR-2/pS2 positive cases (%)* | *Positive tumours, mean proportion positively-stained cells (%)* | *Range* |
|---|---|---|---|---|
| Serous cystadenocarcinoma | 14 | 2 (14%) | 3% | 1–5% |
| Mucinous cystadenocarcinoma | 8 | 6 (75%) | 20% | 14–29% |
| Serous cystadenoma | 14 | 5 (36%) | 18% | 15–24% |
| Mucinous cystadenoma | 11 | 11 (100%) | 37% | 10–71% |

is normally expressed. The factors that control the ectopic expression of this protein are currently unknown.

In human breast cancer at least, expression of the pNR-2/pS2 mRNA or protein is almost entirely confined to a proportion of breast cancers that express oestrogen receptor (Rio *et al.*, 1987; Skilton *et al.*, 1989; Henry *et al.*, 1990; Foekens *et al.*, 1990). This implies that oestrogens regulate the expression of the pNR-2/pS2 gene in breast cancer cells. The question then arises, is this gene regulated by oestrogens acting through the oestrogen receptor in the other malignant tumours in which it is expressed? Oestrogen receptor has been detected in a proportion of ovarian epithelial tumours (Schwartz *et al.*, 1982; Bizzi *et al.*, 1988) and endometrial carcinomas (Palmer *et al.*, 1988). More surprisingly, oestrogen receptor has also been detected in gastric carcinomas (Sica *et al.*, 1984; Tokunga *et al.*, 1986), colorectal carcinomas (McClendon *et al.*, 1977; Sica *et al.*, 1984) and pancreatic carcinomas (Greenway *et al.*, 1981). Furthermore, a favourable response to antioestrogen therapy and other endocrine therapies has been recorded in pancreatic carcinoma (reviewed by Greenway, 1987). Expression of pNR-2/pS2 in normal gastric mucosa and gastric carcinomas has however been reported to be independent of oestrogen receptor expression (Rio *et al.*, 1988*a*; Luqmani *et al.*, 1989) and Wysocki *et al.* (1990) only observed a weak correlation between oestrogen receptor mRNA and pNR-2/pS2 mRNA expression in ovarian epithelial tumours. Unfortunately, as the tumours in this study are an archival series from which only formalin fixed, paraffin embedded material is available and as receptor assays were not performed at the time of resection we are unable to correlate oestrogen receptor and pNR-2/pS2 expression. However in the current series, gender (and consequent gender dependent differences in sex steroid levels) did not show any relationship to the proportion of tumours expressing pNR-2/pS2 (Table I). As the majority of tumours from women were from elderly, postmenopausal women analysis of the effect of menopausal status was not possible.

Other factors may regulate pNR-2/pS2 gene expression in these tumours. The pNR-2/pS2 gene has a complex upstream promoter region that contains enhancer elements responsive to epidermal growth factor, the c-Ha-*ras* oncoprotein, the c-jun protein and a tumour promoter (Nunez *et al.*, 1989). Co-expression of epidermal growth factor and pNR-2/pS2 has been described in intact mucosa at the edge of intestinal ulcers and it has been suggested that in this instance pNR-2/pS2 expression is controlled by epidermal growth factor (Wright *et al.*, 1991). It is also possible that pNR-2/pS2 is expressed constitutively in a proportion of tumours.

The function of the pNR-2/pS2 protein however remains enigmatic. If the pNR-2/pS2 protein is indeed a growth factor it is possible that it may stimulate tumour growth by autocrine means. The biological and prognostic significance of pNR-2/pS2 expression, particularly in non-mammary tumours, remains to be determined. The frequent expression of this protein in diverse human malignancies suggests that pNR-2/pS2 could have a more general role in human neoplasia than hitherto realised.

This work was supported by the Gunnar Nilsson Cancer Research Trust, the North of England Cancer Research Campaign, the Breast Cancer Research Trust, and the Medical Research Council. We thank Mrs R. Brown for technical assistance, Mrs E. Tweedy for secretarial assistance and Mr S. Brabazon for photography. We are indebted to Ms S. Cousen for peptide synthesis. F.E.B. May thanks the Royal Society for a University Research Fellowship.

### References

BERRY, M., NUNEZ, A.-M. & CHAMBON, P. (1989). Estrogen-responsive element of the human pS2 gene is an imperfectly palindromic sequence. *Proc. Natl Acad. Sci. USA*, **86**, 1218.

BIZZI, A., CODEGONI, A.M., LANDONI, F. & 5 others (1988). Steroid receptors in epithelial ovarian carcinoma: relation to clinical parameters and survival. *Cancer Res.*, **48**, 6222.

FOEKENS, J.A., RIO, M.-C., SEGUIN, P. & 5 others (1990). Prediction of relapse and survival in breast cancer patients by pS2 protein status. *Cancer Res.*, **50**, 3832.

GREENWAY, B., IQBAL, M.J., JOHNSON, P.J. & WILLIAMS, R. (1981). Oestrogen receptor proteins in malignant and fetal pancreas. *Br. Med. J.*, **283**, 751.

GREENWAY, B.A. (1987). Carcinoma of the exocrine pancreas: a sex hormone responsive tumour? *Br. J. Surg.*, **74**, 441.

HENRY, J.A., NICHOLSON, S., HENNESSY, C., LENNARD, T.W.J., MAY, F.E.B. & WESTLEY, B.R. (1990). Expression of the oestrogen regulated pNR-2 mRNA in human breast cancer: relation to oestrogen receptor mRNA levels and response to tamoxifen therapy. *Br. J. Cancer*, **61**, 32.

HENRY, J.A., PIGGOTT, N.H., MALLICK, U.K. & 4 others (1991). pNR-2/pS2 immunohistochemical staining in breast cancer: correlation with prognostic factors and endocrine response. *Br. J. Cancer*, **63**, 615.

JAKOWLEW, S.B., BREATHNACH, R., JELTSCH, J.-M., MASIAKOWSKI, P. & CHAMBON, P. (1984). Sequence of the pS2 mRNA induced by estrogen in the human breast cancer cell line MCF-7. *Nucleic Acids Res.*, **12**, 2861.

LUQMANI, Y., BENNETT, C., PATERSON, I. & 4 others (1989). Expression of the pS2 gene in normal, benign and neoplastic human stomach. *Int. J. Cancer*, **44**, 806.

MCCLENDON, J.E., APPLEBY, D., CLAUDON, D.B., DONEGAN, W.L. & DECOSSE, J.J. (1977). Colonic neoplasms: tissue estrogen receptor and carcinoembryonic antigen. *Arch. Surg.*, **112**, 240.

MASIAKOWSKI, P., BREATHNACH, R., BLOCH, J., GANNON, F., KRUST, A. & CHAMBON, P. (1982). Cloning of cDNA sequences of hormone regulated genes from the MCF-7 human breast cancer cell line. *Nucleic Acids Res.*, **10**, 7895.

MAY, F.E.B. & WESTLEY, B.R. (1986). Cloning of estrogen regulated messenger RNA sequences from human breast cancer cells. *Cancer Res.*, **46**, 6034.

NUNEZ, A.-M., JAKOWLEV, S., BRIAND, J.-P., GAIRE, M., KRUST, A., RIO, M.-C. & CHAMBON, P. (1987). Characterization of the estrogen-induced pS2 protein secreted by the human breast cancer cell line MCF-7. *Endocrinology*, **121**, 1759.

NUNEZ, A.-M., BERRY, M., IMLER, J.-L. & CHAMBON, P. (1989). The 5′ flanking region of the pS2 gene contains a complex enhancer region responsive to oestrogens, epidermal growth factor, a tumour promoter (TPA), the c-Ha-ras oncoprotein and the c-jun protein. *EMBO J.*, **8**, 823.

PALMER, D.C., MUIR, I.M., ALEXANDER, A.I., CAUCHI, M., BENNETT, R.S. & QUINN, M.A. (1988). The prognostic importance of steroid receptors in endometrial carcinoma. *Obstet. Gynecol.*, **72**, 388.

PIGGOTT, N.H., HENRY, J.A., MAY, F.E.B. & WESTLEY, B.R. (1991). Antipeptide antibodies against the pNR-2 oestrogen-regulated protein of human breast cancer cells and detection of PNR-2 expression in normal tissues by immunohistochemistry. *J. Pathol.*, **163**, 95.

PRUD'HOMME, J.-F., FRIDLANSKY, F., LE CUNFF, M. & 4 others (1985). Cloning of a gene expressed in human breast cancer and regulated by estrogen in MCF-7 cells. *DNA*, **4**, 11.

RIO, M.C., BELLOCQ, J.P., GAIRARD, B. & 7 others (1987). Specific expression of the pS2 gene in subclasses of breast cancers in comparison with expression of the estrogen and progesterone receptors and the oncogene ERBB2. *Proc. Natl Acad. Sci. USA*, **84**, 9243.

RIO, M.-C., BELLOCQ, J.-P., GAIRARD, B., KOEHL, C., RENAUD, R. & CHAMBON, P. (1988*a*). Expression specifique de gene humain pS2 dans les cancers du sein. *Biochimie*, **70**, 961.

RIO, M.C., BELLOCQ, J.P., DANIEL, J.Y. & 5 others (1988*b*). Breast cancer-associated pS2 protein: synthesis and secretion by normal stomach mucosa. *Science*, **241**, 705.

SCHWARTZ, P.E., LIVOLSI, V.A., HILDRETH, N., MACLUSKY, N.J., NAFTOLIN, F.N. & EISENFELD, A.J. (1982). Estrogen receptors in ovarian epithelial carcinoma. *Obstet. Gynecol.*, **59**, 229.

SICA, V., NOLA, E., CONTIERI, E. & 7 others (1984). Estradiol and progesterone receptors in malignant gastrointestinal tumours. *Cancer Res.*, **44**, 4670.

SKILTON, R.A., LUQMANI, Y.A., MCCLELLAND, R.A. & COOMBES, R.C. (1989). Characterisation of a messenger RNA selectively expressed in human breast cancer. *Br. J. Cancer*, **60**, 168.

STERNBERGER, L.A., HARDY, P.H., CUCULIS, J.J., & MEYER, H.G. (1970). The unlabelled antibody method of immunohistochemistry: preparation and properties of soluble antigen-antibody complex (horseradish peroxidase: antiperoxidase) and its use in the identification of spirochaetes. *J. Histochem. Cytochem.*, **18**, 315.

TOKUNGA, A., NISHI, K., MATSUKURA, N. & 5 others (1986). Estrogen and progesterone receptors in gastric cancer. *Cancer*, **57**, 1376.

WRIGHT, N.A., POULSON, R., STAMP, G.W.H. & 6 others (1991). Epidermal growth factor (EGF/URO) induces expression of genes encoding regulatory peptides in damaged human gastrointestinal tissues. *J. Pathol.*, **162**, 279.

WOOD, G.S. & WARNKE, R. (1981). Suppression of endogenous avidin-binding in tissues and its relevance to biotin-avidin detection systems. *J. Histochem. Cytochem.*, **29**, 1196.

WYSOCKI, S.J., HAHNEL, E., MASTERS, A., SMITH, V., MCCARTNEY, A.J. & HAHNEL, R. (1990). Detection of pS2 messenger RNA in gynaecological cancers. *Cancer Res.*, **50**, 1800.